# Tumor-related interleukins: old validated targets for new anti-cancer drug development

**DOI:** 10.1186/s12943-017-0721-9

**Published:** 2017-09-19

**Authors:** Sarra Setrerrahmane, Hanmei Xu

**Affiliations:** 1 The Engineering Research Center of Peptide Drug Discovery and Development, China Pharmaceutical University, Nanjing, Jiangsu 210009 People’s Republic of China; 2 State Key Laboratory of Natural Medicines, Ministry of Education, China Pharmaceutical University, Nanjing, Jiangsu 210009 People’s Republic of China

**Keywords:** Cancer, Tumor microenvironment, Immune cells, Cytokines, Anti-cancer

## Abstract

In-depth knowledge of cancer molecular and cellular mechanisms have revealed a strong regulation of cancer development and progression by the inflammation which orchestrates the tumor microenvironment. Immune cells, residents or recruited, in the inflammation milieu can have rather contrasting effects during cancer development. Accumulated clinical and experimental data support the notion that acute inflammation could exert an immunoprotective effect leading to tumor eradication. However, chronic immune response promotes tumor growth and invasion. These reactions are mediated by soluble mediators or cytokines produced by either host immune cells or tumor cells themselves. Herein, we provide an overview of the current understanding of the role of the best-validated cytokines involved in tumor progression, IL-1, IL-4 and IL-6; in addition to IL-2 cytokines family, which is known to promote tumor eradication by immune cells. Furthermore, we summarize the clinical attempts to block or bolster the effect of these tumor-related interleukins in anti-cancer therapy development.

## Background

Early in 1863, Virchow postulated cancer proliferation at sites of chronic inflammation and infection. His conclusion was based, in part, on the hypothesis that some classes of mediators causing the inflammation can enhance tumor cells proliferation, in another part on the evidences revealing the presence of leukocytes in neoplastic tissue [1]. Several experimental and epidemiological data came to support this contention. Examples include increased incidence of virus-associated cancers, liver and pancreas cancer caused by alcohol induced inflammation and smoking- related lung inflammation and carcinoma [2–5]. Other data suggested that sporadic or inherited genetic mutations in some critical genes regulating cell cycle, programmed cell death or differentiation and adhesion might be the actual cause of tumorigenesis. Chronic inflammation and cytokines production favor selection of additional features in initiated cells, which may be the promotor of malignant transition [6, 7].

**Fig. 1:**
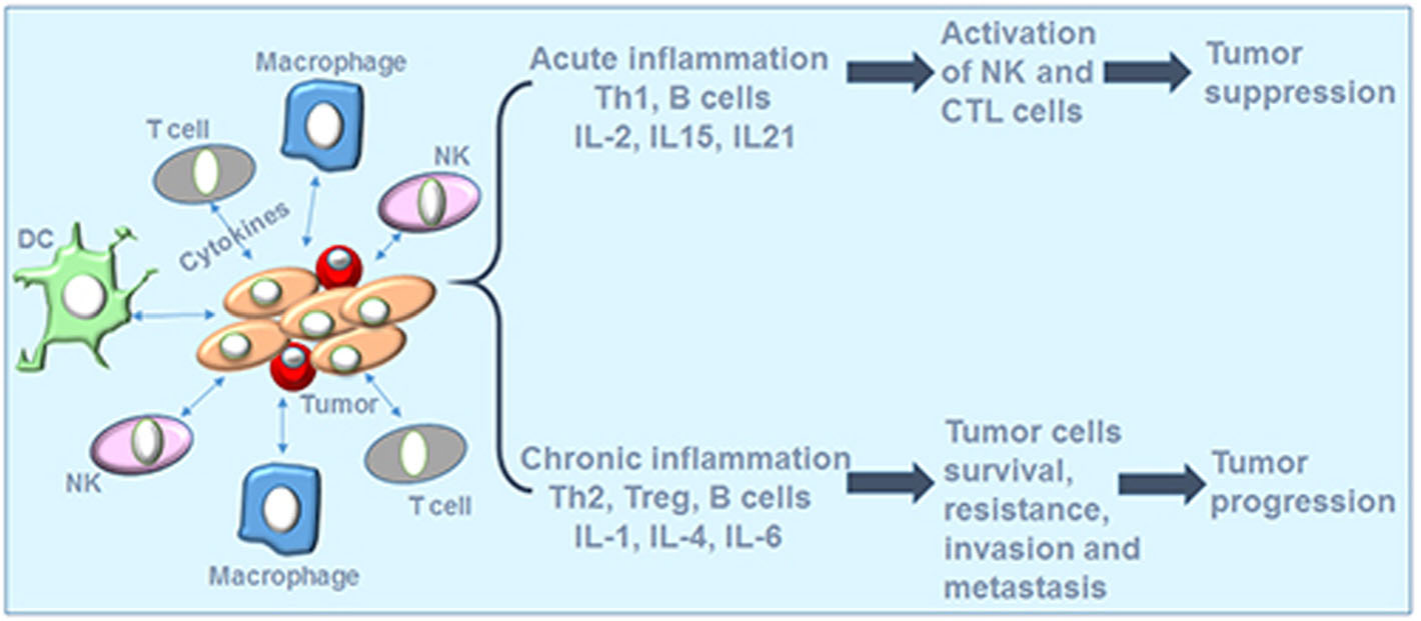
Interactions between tumor cells and infiltrating immune cells in the tumor microenvironment Cytokines secreted by both tumor and immune cells could either induce tumor suppression or promote tumor progression. Acute inflammatory response through IL-2, IL-15, and IL-21 activates antimpetumor immune response by activating NK and CTL cells. However, chronic inflammation results in tumor cells escape from immune response through the action of different mediators especially, IL-1, IL-4 and IL-6.

The tumor microenvironment is rich in a variety of immune cells, composed of both myeloid (innate immunity) and lymphoid (adaptive immunity) lineages [8–10]. The former involves macrophages, granulocytes, mast cells, dendritic cells (DCs), and natural killer (NK) cells. Contrary to what was thought, not all these leukocytes represent an attempt by the host to eradicate transformed neoplastic cells. Only some are [11], and all other classes may support tumor growth, invasion, metastasis, and escape from the host immune response and conventional anti-cancer therapy [12, 13]. On the other hand, adaptive immunity is generally represented by B and T lymphocytes. Acute activation of B cells may play a role in eradicating early neoplastic cells, or inducing tumor regression via the secretion of antigen-specific immunoglobulins [14]; meanwhile, the chronic activation of B cells may paradoxically play a role in potentiating cancer development. Moreover, cytotoxic T lymphocytes (CTLs) recruited in acute tumor-directed immune responses, appear to protect against tumor development [15, 16], whereas the immune responses involving chronic activation of humoral immunity and infiltration of Th2 cells, result in the promotion of tumor development and disease progression [16]. As shown in Fig. 1, the regulation of such immune responses is mediated by the cytokines secreted to initiate or to weaken the host antitumor immunity, [17, 18] .

In chronic inflammation, tissue homeostasis is dramatically perturbed. Tissue-resident macrophages and mast cells locally secrete soluble factors such as cytokines, chemokines, bioactive mediators, and matrix-remodeling proteins that recruit additional leukocytes from the circulation into damaged area [19], this contributes to the development of most human solid tumors. Recently, a growing body of literature claimed that cytokines are not only produced by the immune cells present in the tumor microenvironment, the autocrine origin of some inflammatory cytokines, especially IL-1, IL-4 and IL-6, was observed in large range of solid tumors [20–22]. Cancer cells not only express the cytokine, but also over expresses the related receptor to use it and escape from the immune response [23, 24]. The secreted molecules could also serve as messengers in the cross talking between cancer cells and tumor infiltrating immune cells.

In short, cytokines are a key part in combating cancer and understanding their mechanisms of expression, activation and action might be of substantial benefit in developing anti-cancer drug either for direct targeting the tumor as a mono therapy or as an adjuvant in combination with other therapeutic agents. The most validated cytokines involved in cancer progression are IL-1, IL-4, IL-6, while IL-2 has been the most understood anti-inflammatory cytokines used to boost the host immunity against cancer.

The present review summarizes the most known cancer-related cytokines IL-2, IL-1, IL-4 and IL-6 as regulators of carcinogenesis and cancer maintenance or eradication. Moreover, it describes the current knowledge on the intracellular signaling pathways downstream of their receptors and highlights the recent experimental data indicating that therapeutically targeting these diverse interleukins by either neutralizing and/or bolstering their specific bioactivities may provide a platform for the design of new therapeutics for cancer immunotherapy.

## Il-2

Being a member of the γ chain cytokine family, and a key cytokine in regulating the survival, proliferation, and differentiation of activated T cells and NK cells [25], IL-2 is used to activate the immune system of cancer patients as one of the most important areas of current cancer immunotherapy research. The results obtained from IL-2 immunotherapy proved for the first time that the immune system could completely eradicate tumor cells under certain circumstances [26–29]. Results of complete remission and long disease-free post treatment period have been reported after administration of high doses, but also low doses, to patients with melanoma and renal cell carcinoma. [30], and lead to the approval of IL-2 immunotherapy for the treatment of metastatic renal cell carcinoma and later for metastatic melanoma [31].

**Fig. 2:**
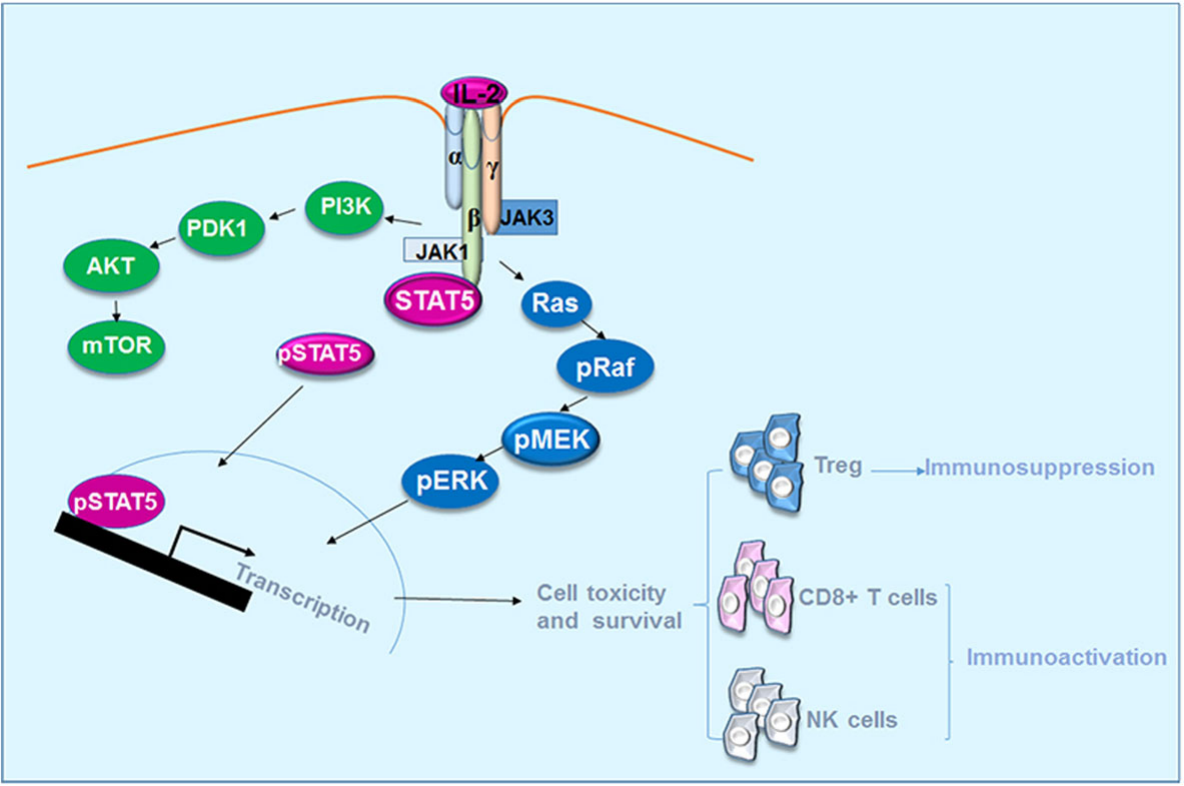
Signaling pathways of IL-2. IL-2 activates JAK-STAT, PI3K and MAPK signaling pathways upon its engagement in the specific receptor complexes. The signaling pathway results in the activation and survival of Treg, CD8+ T cells and NK cells

IL-2 receptor (IL-2R) is composed by three subunit α, β and γ. IL-2Rα is mainly expressed by immune cells including T regulatory cells (Treg), and by activated CD4^+^ and CD8^+^ T cells, B cells, CD56^hi^ NK cells, mature dendritic cells (DCs), and endothelial cells [32–35] at low level. The IL-2Rβ is expressed by multiple lymphoid populations such as Treg, memory CD8^+^ T cells, NK cells, monocytes and neutrophils [25, 36, 37], while the IL-2Rγ subunit is expressed mainly by hematopoietic cells. IL-2 has a greater binding affinity toward IL-2Rαβγ trimeric complex than to the single or the heterodimer IL-2R subunit [38–40]. High affinity binding of IL-2 leads to the recruitment and activation of Janus family tyrosine kinases (JAK1 and JAK3) starting three major downstream signaling pathways, the signal transducer and activator of transcription (STAT5A, STAT5B) signaling pathway, the phosphoinositide 3- kinase (PI3K-AKT) signaling pathway, and the mitogen-activated protein kinase (MAPK) signaling pathway [25] (Fig. 2).

IL-2 is a pivotal immunoactivator agent; it promotes T cell clonal expansion and effector differentiation. In the early phase of immune response, IL-2 acts by promoting the differentiation of naive CD8^+^ T cells into effector and memory effector cells [41]. Similar to T cells, the activation and cytolytic activity as well as the homeostasis and survival of NK cells are highly controlled by IL-2 [42]. In activated T cells and NK cells, it lead to the upregulation of perforin, granzyme B and cytokines production including IFN-γ and TNF-α.

Moreover, because Treg cells are the only T cells that highly express α subunit of IL-2R, they present a high affinity to IL-2, which binds to IL-2Rαβγ complex, and induces the development and maintenance of the immunosuppressive Treg population in the body, preventing the development of autoimmunity [43]. This immunosuppressive effect has been mainly attributed to expanding peripheral CD4^+^CD25^+^Foxp3^+^ Treg, by inducing the expression of the *Foxp3* gene in natural (nTreg) and induced Treg (iTreg) [16, 44]. Both nTreg and iTreg can efficiently suppress the function of CD4, CD8, and NK cells, and lead to the failure to generate an efficient immune response. The persistence of high level of CD4^+^CD25^+^Foxp3^+^ Treg in the serum of patients treated with IL-2, was associated with poor clinical response [42, 45]. It has also been shown that IL-2 drives T cells and NK cells quiescence and apoptosis. It acts in the terminal differentiation of CD8^+^ T cells, and limits T cell numbers by the downregulation of γc receptor and Bcl-2 expression, thereby rendering them more susceptible to apoptosis.

## Application of IL-2 in cancer therapy

IL-2 is commonly used in cancer therapy as immunostimulating agent to compensate the immunosuppressive cytokines secreted by cancer cells. To achieve the therapeutic goal, IL-2 has been tested alone or in combination with chemotherapy, radiotherapy, vaccine, regimens and cytokines in several clinical trials (Table 1).

**Table 1:** Summary of clinical trials utilizing IL-2 in anti-cancer immunotherapy

Treatment	Cancer type	Overall response rate	Side effects	Ref.
IL-2 monotherapy	Metastatic Melanoma, Renal cell carcinoma	6.6% complete response 19%	Expected IL-2 related side effects	[46]
IL-2 plus IFN-α2b	Metastatic Renal Cancer	9.9%	Expected IL-2 related side effects	[187]
IL-2 plus IFN-α2b plus chemotherapy	Metastatic Melanoma	19.5%	Chemotherapy related toxicity, some of IL-2 related side effects	[188]
IL-2 plus Ipilimumab (anti-CTLA-4 antibody)	Stage IV Melanoma	22%	Expected IL-2 related side effects, Ipilimumab related autoimmune toxicities	[47]
IL-2 plus gp100 vaccine	Stage III Melanoma	16%	Some IL-2 related toxicity	[189]
IL-2 plus IL-2 expanded TILs	Melanoma	48.4%	Expected IL-2 related side effects	[190]
IL-2 plus LAK cells Darleukin (L19IL2) Daromun (L19IL2 + L19TNF)	Melanoma Renal Cell Carcinoma (RCC) Colorectal cancer, non-Hodgkin’s Lymphoma, Stage IIIB/C melanoma Stage IIIB/C and IVM1a melanoma	21% 35% No observed response No observed response 53.9%, 25% complete remission 55%, 5% complete remission	Expected IL-2 related side effects Limited IL-2 related side effects Limited IL-2 related side effects	[45, 191] [192] [193]

In a study done by Rosenberg and his collaborators, 409 patients with either metastatic melanoma or renal carcinoma, were treated with a high-dose of IL-2 (720,000 lU/kg), a complete response was observed in 8.1% patients, and a partial response in 9% patients. 82% of completely responding patients remained disease free for 39 to more than 148 months from the onset of treatment [46]. Additionally, it has been reported that IL-2/IL-2R interaction has a critical impact on CTLA-4 antagonist antibodies effects. IL-2 and its receptor subunits (IL-2Rα and IL-2Rβ) are critical for T cell-dependent control of tumor progression induced by CTLA-4 blockade, which could change the functional profile of the suppressive CD4^+^Lag3^+^ T cells to the regulatory Foxp3^−^ T cells, tobecome the major source of intratumoral IL-2 [47].

Besides its application in melanoma and renal cell carcinoma, the possibility of IL-2 utilization in other solid tumor types was tested. Hopeful results were obtained in colorectal and non-small cell lung cancer (NSCLC) treatment. Furthermore, results of a meta-analysis of randomized studies support the use of IL-2 in combination with conventional chemotherapy in solid tumors, especially for colorectal cancers. Also, low doses of IL-2 were used (200 mIU) as an adjuvant therapy with dendritic cells (DCs) vaccination in a clinical trial for recurrent ovarian cancer treatment. The result showed a good tolerance by the treated patients, associated with induction of tumor-related immunity, and long-term clinical responses against ovarian cancer [48].

In addition to its therapeutic applications, IL-2 became a key cytokine used in the ex vivo expansion of T cells isolated from tumors, and applied in the transfer of highly expanded tumor infiltrating lymphocytes (TILs) for melanoma [49, 50]. More recently, IL-2 has also been used for adoptive cell therapy to expand peripheral blood T cells transduced with antigen-specific T-cell receptors (TCRs) [51, 52] and chimeric antigen receptors (CARs) [53].

IL-2 still has several drawbacks that limit its application clinically, mainly a very short half-life and high treatment related toxicity. Several approaches are ongoing to overcome these limitations and extend the use of IL-2 in cancer immunotherapy; Zhu et al. have fused IL-2 with IgG2 Fc fragment to generate an extended half-life form. Preclinical studies on a melanoma mice model showed that IL2-Fc exerts a synergetic action with different antitumor antibodies, by promoting NK Cell and CD8^+^ T cell activation and consolidating with the antibodies antitumor therapy [54].

Otherwise, most of the clinical trials required a systematically high dose of IL-2 to achieve the therapeutic benefit. Indeed, this high dose resulted in harmful side effects particularly hypotension, vascular leak syndrome (VLS), pulmonary edema, and heart toxicities. Several approaches aimed to limit the side effects of IL-2 systemic therapy by targeting IL-2 to the tumor milieu and restricting its effects on the other organs. Chaurasiya and his research team have recently reported the use of a nonreplicating adenovirus vector for hIL-2 expression in breast cancer cells under the control of an engineered human mammaloglobin promoter/enhancer (MPE2). The results revealed a high expression of IL-2 in breast cancer cells, while lower expression level was observed in human and murine normal cells. Animal model showed a significant delay in tumor growth, with negligible liver toxicity using the MPE2-controlled vector compared to a CMV controlled vector. In this approach, the virus-mediated reaction helped the immune system recognize the cancer cells, while the expressed cytokine boosted the antitumor effect [55]. In another study, a tumor-targeted replication-competent virus (oncolytic virus) carrying the genes encoding for IL-2 and TNF-α, was used for IL-2 delivery to the tumor site. The virus was double mutated to render its replication specific and selective for cells defective in the retinoblastoma/p16 pathway observed in most of the cancer cells. The administration of the adenovirus to SCID mice and immunocompetent Syrian hamsters with melanoma resulted in the expression of biologically active cytokines and a synergy was observed with TILs combination. This treatment led to 100% treatment of the experimental animals and their protection from tumor rechallenge, a clinical trial is in progress to study the utility of the oncolytic adenovirus in human cancers [56]. L19-IL-2 (Darleukin), a diabody with two human IL-2 molecules genetically fused to the C-terminus of each scFv domain, and L19-IL-2 + L19-TNF (Daromun) by associating TNF therapy to the first molecule, are two other targeted immunocytokines under development. The two drugs showed promising effects in Phase II and III when administrated as an intratumoral injection for the treatment of melanoma. (NCT01253096, NCT02076633 and NCT02938299) [57].

The other drawback of IL-2 therapy is that it preferentially induces the expansion of CD4^+^CD25^+^Foxp3^+^ Treg cells. These cells prevent antitumor lymphocyte activity and tumor eradication, and was found to be correlated with poor clinical response, numerous IL-2 mutants (superkines) are under development to replace the native IL-2 and limit this undesired effect. F42 K and R38A, two IL-2 superkines under development, having an altered IL-2Rα binding domain which affects their binding affinity to IL-2αR highly expressed in Treg cells, while preserving an affinity similar to that of native IL-2 to the IL-2Rβγ expressed on effector T cells and NK cells. Some results showed that these IL-2 mutants have less effects in stimulating Treg cells and could activate lymphokine-activated killer (LAK) cells without the production of high levels of pro-inflammatory cytokines which induce the systemic toxicity [58].

Finally, the factors that constrain the curative potential of IL-2 to only a small percentage of patients is still unknown and need to be further explored.

## Other IL-2 family cytokines

IL-15 and IL-21 belong to IL-2 family, emerged as key cytokines playing with IL-2 a synergistic and unique role in the antitumor immune response regulation. Several pre-clinical and clinical research investigated the effect of these cytokines in cancer immunotherapy. However, their mechanisms of action are still not clearly defined.

IL-15 shares many structural and functional similarities with IL-2. It binds to the same cell surface complex in a very similar way, and activates the same downstream signaling pathway [59, 60]. It has been reported that IL-15 may have some overlapping signaling with the T-cell receptor complex (TCR) [61]. IL-15 exhibits less expanding Treg efficiency than IL-2. However, it promotes more memory maintenance of CD8^+^ T cells, and induces more NK cells survival [62]. Because of the grave side effects of IL-2, application of IL-15 in anti-cancer immunotherapy has been extensively investigated in vitro and in vivo*.* Several results proved the potential of IL-15 therapy in mediating tumor regression [63]. IL-15 effect was found to be largely dependent on the enhancement of NK cell cytotoxicity [63] and the activation of CD8^+^ T cells. Recent preclinical studies have supported the combination of IL-15 with CD19 specific CAR-T cells, anti-PD-1 or anti-CTLA-4 therapy [64]. Clinical trials are ongoing to test the application of IL-15as single therapy for advanced solid tumors such as melanoma, kidney cancer, non-small cell lung cancer, and squamous cell head and neck cancer (NCT01727076) and for lymphoma (NCT01572493); or in combination with haploidentical donor NK cells for leukemia treatment (NCT01385423). Other clinical trials showed the safety and the feasibility of using IL-15-expanded CD3/CD19 cells [65] or cytokine-induced killer cells (CD3^+^CD56^+^ T cells) to treat leukemia patients [66].

IL-21 is a key regulator of NK cell differentiation later added to IL-2 cytokines family. IL-21 binds specifically to IL-21R activating STAT, PI3K-AKT, and MAPK pathways, with more preference to phosphorylates STAT3 and STAT1 rather than STAT5A and STAT5B [67]. IL-21 has been found to promote a strong proliferation of NK cell and expression of effector molecules in NK cells, while being a poorer driver of T-cell expansion. A preclinical study using an IL-21 plasmid expression system in melanoma and fibrosarcoma treatment, showed potent antitumor effect and increased survival rate of tumor-bearing mice [68]. Further research showed more efficacy of CD8^+^ T cells pre-cultured with IL-21 than for those pre-cultured with IL-2 [69]. Other studies revealed that adoptive transfer of IL-21-pre-cultured CAR-T cells has also improved the function of T cells and inhibited proliferation of CD19^+^ B-cell malignancy in mice [70]. In addition, co-administration of IL-21 and IL-15 enhanced the expression of IFN-γ by CD8^+^ T cells and induced melanoma tumor regression. In clinic, phase I and II trials for IL-21 as single-agent, revealed a modest response of patients with melanoma [71]. Ongoing clinical trials are testing IL-21 combination therapy with sorafenib (NCT00389285), sunitinib (NCT00617253), ipilimumab (NCT014890 59) or anti-PD-1 (NCT01629758) to treat patients with metastatic melanoma and renal cell carcinoma.

## Il-1

IL-1 is a central pro-inflammatory cytokine and important mediator of innate and adaptive immunity. IL-1 exists in two form, IL-1α and IL-1β, which trigger very similar effects in terms of signal transduction, activation of intracellular signaling pathways, and regulation of gene expression. For instance, the two isoform mainly differ in their synthesis and compartmentalization within the cell or the tumor microenvironment. IL-1α is located in both the nucleus and cytoplasm, and its active form exists as membrane bound or rarely as mature secretory forms in immune cells (macrophages and monocytes). In particular, the membrane bound form requires only activation of NFκB, whereas the secretory IL-1α needs additional activating stimuli from the inflammasome and caspase 1 [44, 72]. IL-1β is synthesized as a precursor protein pro-IL-1β in the cytoplasm, and is uniquely active as a secreted form after cleavage by caspase 1 [73].

**Fig. 3:**
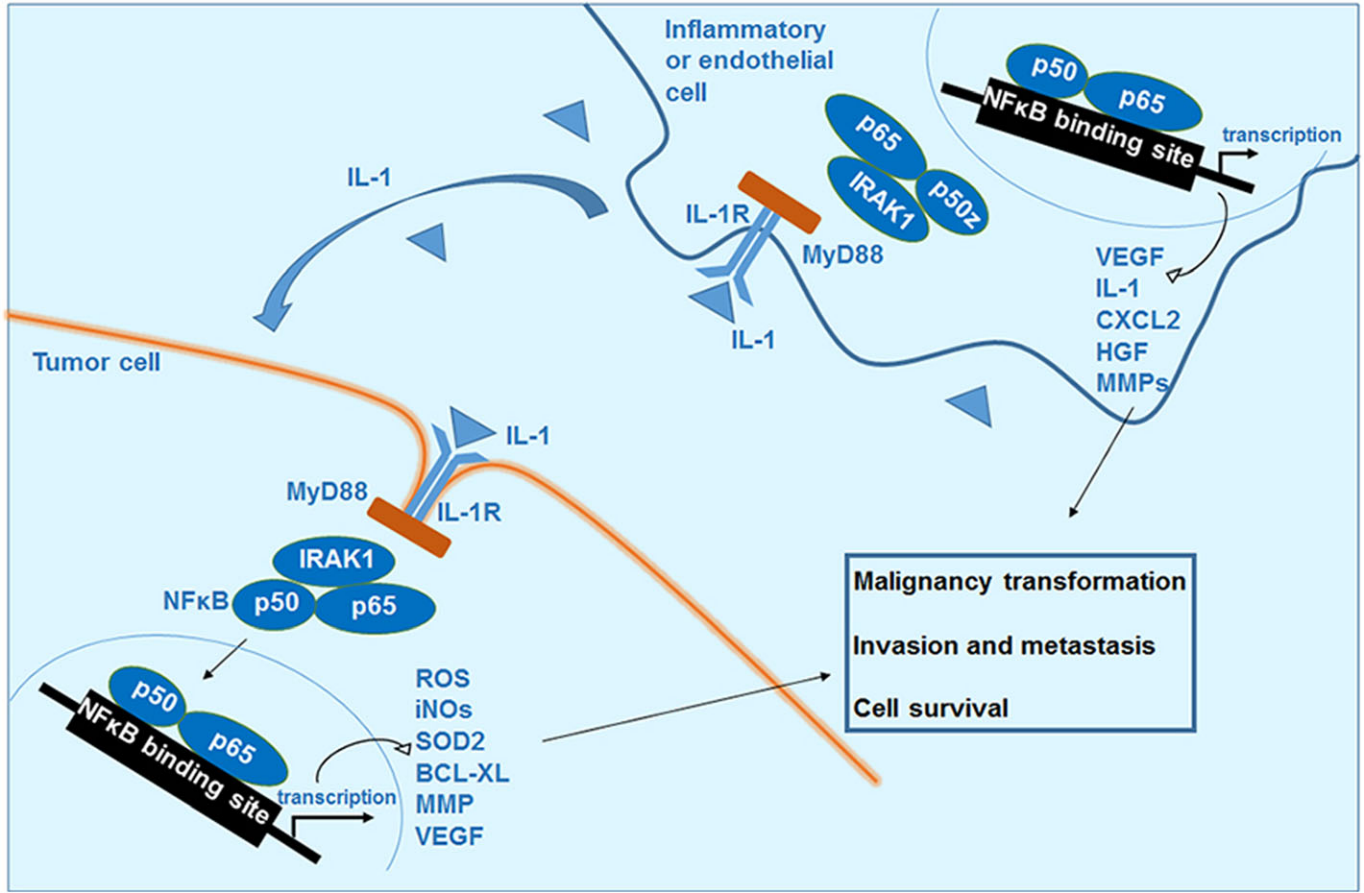
the role of IL-1 in cell carcinogenesis and tumor metastasis. The interaction of IL-1 with its receptor activates the NFκB in different cell types. In cells involving in the malignancy transformation, it upregulates the gene of reactive oxygen species, nitrogen oxide intermediates and anti-apoptotic factors. In the inflammatory or endothelial cells it upregulates the genes expressing cytokines and factors supporting cells survival and invasion

IL-1 receptors (IL-1Rs) are expressed on the majority of cell types including T cells, myeloid cells, fibroblasts, and cancer cells. IL-1 has affinity to two types of receptors: IL-1R1 and IL-1R2. IL-1R1 is a signaling capable receptor whereas IL-1R2 is required for binding the soluble IL-1 and making its complex with IL-1R1. Signaling from IL-1 via surface IL-1R could be initiated by the inflammation factors secreted during a bacterial infection, or by tissue-damage products released from necrotic cells in response to trauma or ischemia, in the case of sterile inflammation notably cancer. The downstream signaling pathway is initially triggered by the activation of NFκB as a regulator of its immature form expression, and accomplished by recruiting the MyD88 adaptor molecule, which in turn attracts and activates the protein kinase IL-1 receptor-associated kinase (IRAK1), resulting in the activation of several kinases from MAPK family, as well as nuclear genes especially via the NFκB and subsequent induction of inflammatory response [74] (Fig. 3).

## IL-1 and cancer

IL-1β is one of the most potent pro-inflammatory cytokines influencing the growth and invasion of nearly all types of tumor cells due to its abundance in tumor site and patients serum in contrast to IL-α, which is mainly membrane-associated and less diffusible in tumor microenvironment [75]. High levels of IL-1β in tumor and serum are associated with higher tumor grade and increased invasion in breast, pancreatic cancer and myelogenous leukemia, and are also correlated with poor patient outcome [76–80]. IL-1 participates in all phases of tumor development from the generation of malignant cells and carcinogenesis, to the cancer invasion and dissemination. Also, it patterns the interactions of the malignant cells with the host’s immune system.

The role of IL-1β in the process of carcinogenesis may be attributed to mutagenesis induction by activating the infiltrating phagocytes or fibroblasts, and the target cells, to produce mutagenic reactive oxygen species (ROS) or nitrogen oxide intermediates (iNOs). As reported before, using an animal model of chemical carcinogenesis induced by 3-methylcholantrene (3-MCA) [81], fibrosarcomas development in mice deficient in IL-1β was very slow after a prolonged lag period, and only in parts of the animal. However, tumor development was more rapid in wild type and IL-1α deficient mice. Another evidence of IL-1 contribution in cell carcinogenesis was reported by Chen et al., they revealed that IL-1β upregulated the intracellular ROS level and consequently, the expression of glutaredoxin 1 (Grx1) in oral squamous cancer cells (OSCC). Grx1 expression was associated with the malignant transformation process in vivo [82].

Additionally, IL-1 may enhance the invasiveness of already existing tumor cells by the induction of inflammatory molecules, such as matrix metalloproteinase (MMPs), vascular endothelial growth factor (VEGF), heparanase, chemokines, and integrins on the malignant cells and endothelial cells, or by switching on the angiogenesis leading to tumor dissemination and metastasis. An experimental model with murine Lewis lung carcinoma cells transduced with a retroviral vector expressing IL-1β, showed that cells infected with human IL-1β expressing vector grew rapidly in mice despite the lack of a difference in the cell growth in vitro. This rapid in vivo growth pushed the authors to analyze the tumor stroma, and they found that it is associated with hyper neovascularization induced by several angiogenic factors secreted from tumor cells and from stromal cells in the tumor milieu. Their investigation proved that transduced cells secreted 2-fold the amount of VEGF, more than 10-fold the amount of macrophage-inflammatory protein-2 (CXCL2), and more than 4-fold higher concentration of hepatocyte growth factor HGF, which is mainly derived from stromal fibroblasts and infiltrating cells [83]. Moreover, treatment of the lung epithelial cell line A549 with IL-1β under normoxia was shown to induce a markedly increase in HIF-1α levels, which promotes tumor angiogenesis and invasion [84].

Further investigations on prostate cancer using a mouse model, found that overexpression of IL-1β in non-metastatic prostate cancer cells promoted bone metastasis, whereas its knockdown impaired bone invasion by metastatic cells. Membranous overexpression of E-cadherin ad β-catenin after stimulation with IL-1 was significantly associated with prostate cancer metastasis [85].

Jin et al. reported that IL-1β is highly expressed by several triple-negative breast cancer cell lines (TNBC) than non-TNBC. This result was in correlation with other study on human tissue extracts which promulgating a higher IL-1β levels in invasive breast carcinoma compared to non-invasive tumors [76]. The elevated level of IL-1β has been shown to upregulate osteoprotegerin (OPG) expression in the breast cancer cell lines MCF-7 and MDA-MB-231 by activation of p38 and p42/44 MAPK signaling pathway [86]. OPG is a secreted member of the tumor necrosis factor (TNF) receptor super-family, prominently known for its inhibition of TNF-related apoptosis-inducing ligand (TRAIL) mediated apoptosis in vitro [87, 88]. Macrophages are the source of exogenous IL-1β, their co-culture with breast cancer cell lines enhances OPG expression in breast cancer cells.

Mitsunaga et al. later proved that IL-1β increases the invasiveness of TNBC cells in vitro [89, 90] through MMP-3 induction as well as an IL-1β auto-amplification loop. In the same case, IL-1β and its receptor IL-1R1 are upregulated in breast cancer cells that metastasize to bone comparing with non-metastasis cells. Blocking IL-1R1 and 2 with Anakinra (a recombinant IL-1Ra) reduced the metastasis of tumor cells to bone [91].

Recent studies reported that IL-1β can induce prostate cancer stem-like cells proliferation through the upregulation of the pro-survival scaffold protein, Sequestome-1 (SQSTM1/p62) and the repression of androgen receptor accumulation in a relatively small cell subpopulation within various prostate cancer cell lines. Consequently inducing stem cell marker mRNA and protein accumulation, suggesting that IL-1β alters prostate cell fate and supports the notion that inflammation contributes to prostate cancer initiation and progression [92].

Finally, the carriage of IL-1β genetic polymorphism conferred greater risk of a hypochlorhydric, atrophic response to gastric infection with *Helicobacter pylori*, which in turn predisposes to noncardia gastric adenocarcinoma but not to cardia or esophageal cancers [7, 93]. It has been reported that IL-1β is a potent activator of NF-κB which could inhibit cell apoptosis in gastric cancer. IL-1β is also able to directly induce DNA methylation, which may link inflammation-induced epigenetic changes and promote the development of gastric diseases [94, 95].

## Anti-IL-1 therapy in cancer

Currently, IL-1 blocking therapeutics are successfully used in various chronic and inflammatory diseases. The most used IL-1 blocker is “anakinra”, a recombinant form of IL-1R antagonist (IL-1Ra), applied in the treatment of various inflammatory diseases such as rheumatoid arthritis, infections and type 2 diabetes. In addition, the IL-1β antagonist antibody ACZ (canakinumab) has been approved for treating cryopyrin-associated periodic syndromes (CAPS). Finally, Rilonacept, a construct of two extracellular chains of the IL-1 receptor complex (IL-1RI plus IL-1RCcP), fused to Fc segment of IgG has been also approved for CAPS treatment [96, 97].

The clinical application of these agents in cancer treatment is still very limited, and only one clinical trial has been cited in clinicaltrials.gov for anakinra in melanoma treatment, though no results are available from it. It is important to notice that no organ toxicity has been reported for these blockers, and their use was tolerated by the patients, making their use potentially possible for cancer treatment to reduce angiogenesis and inhibit tumor metastasis. The best protocol may be the combination of anakinra with low dose of anti-VEGF agent, in addition to the standard chemotherapy to increase the antiangiogenic effect without increasing VEGF-related toxicity.

## Il-6

IL-6, a multi-functional cytokine in immunity regulation, was originally identified as T cell-derived lymphokine that acts on activated B cells, and induces their final maturation into antibody producing cells. In addition, IL-6 can target resting T cells and induces the proliferation and the differentiation of cytotoxic T cells in the presence of IL-2 [98].

**Fig. 4:**
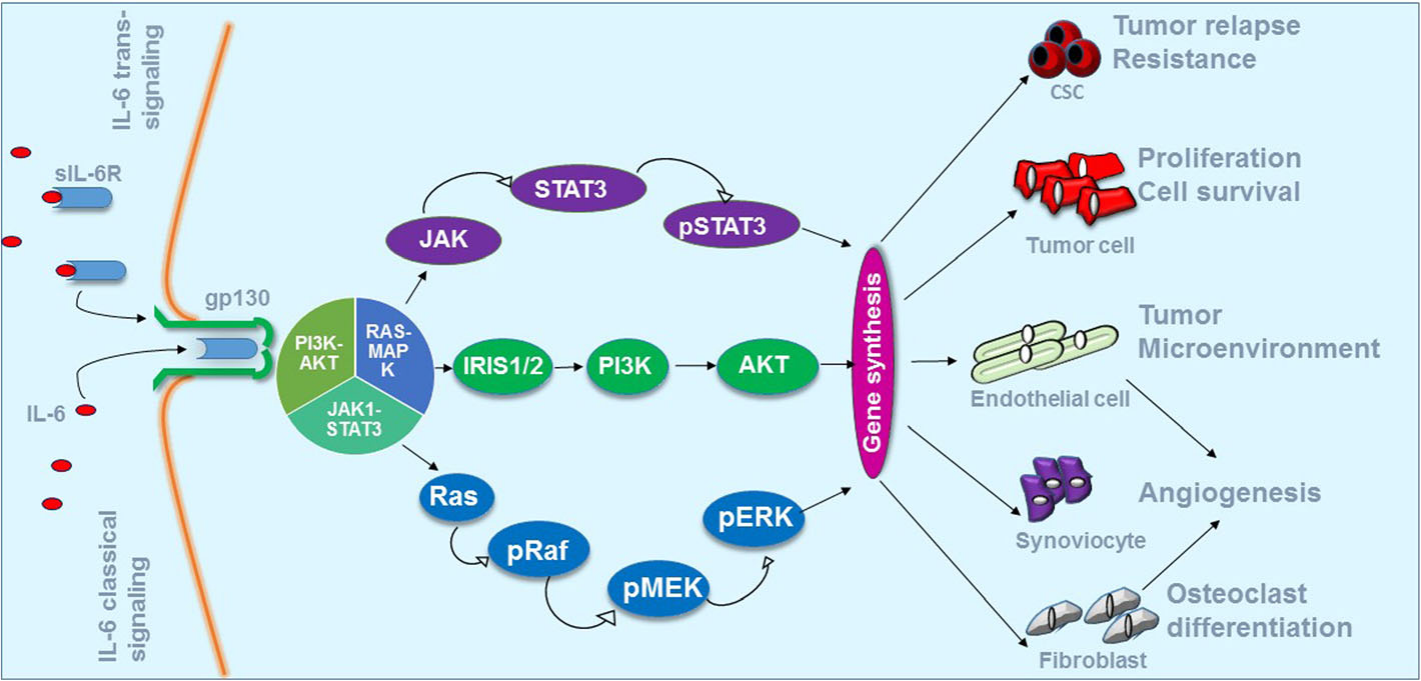
Major IL-6 signaling pathways and their roles in cancer evolution. The classical signaling is mediated by the membrane-associated IL-6R, whereas the trans-signaling by the soluble form sIL-6R. gp130 is ubiquitously expressed on a large range of cell types, thus accounting for the pluripotent activities of IL-6 via the trans-signaling mechanism. Both classical and trans-signaling pathways activate the identical intracellular pathways including JAK1–STAT3, RAS–MAPK, and, PI3K–AKT The proliferation and survival of tumor cells are promoted by IL-6 through both paracrine and autocrine mechanisms. After activation by IL-6, various types of cells, such as tumor-associated macrophages (TAM), myeloid derived suppressor cells (MDSCs), and endothelial cells, are involved in the development of pro-inflammatory and metastatic tumor microenvironment. In addition, IL-6 supports the generation and the survival of cancer stem cells (CSC), induces collagen production by fibroblasts, and promotes the differentiation of osteoclasts and augments angiogenesis.

Two signaling pathways have been described for IL-6: a classical pathway through its cellular receptor, and a trans-signaling pathway through a soluble IL-6-receptor-α (sIL-6R) found in serum, which forms a complex with IL-6 and binds any cell expressing the receptor subunit glycoprotein gp130. The second pathway has been found to be implicated in most of the deleterious effects of IL-6 in chronic inflammatory diseases and cancer [20]. IL-6 binding to IL-6R activates three major signaling pathways, mainly the Janus tyrosine family kinase-signal transducer and activator of transcription (JAK-STAT3) pathway, where JAK phosphorylates and activates the transcription factor STAT3. The phosphorylated STAT3 will then translocate to the nucleus and initiates transcription of its target genes, such as HIF-1α, MMP-2 and -9, BCL-X, BCL-2 and VEGF [99]. IL-6 also activates the ERK1/2-MAPK signaling pathway where JAK phosphorylates SHP2 (Src homology 2 domain containing tyrosine phosphatase 2), which in turn leads to the successive activation of a cascade of events, Ras, Raf, mitogen-activated protein kinase (MEK) and at last ERK. Finally, IL-6 can activate signal transduction through the PI3-K signaling pathway, which recruits the protein kinase Akt to the plasma membrane and binds it. Phosphorylated Akt translocates to the nucleus and other subcellular components, where it regulates the anti-apoptotic and the proliferative processes [100] (Fig. 4).

## IL-6 and cancer

In the physiological situations, IL-6 is secreted by macrophages in response to specific microbial molecules. Aberrantly, elevated IL-6 and IL-6 mRNA levels were observed in the serum of cancer patients and have been associated with poor clinical outcomes [101, 102]. In addition, elevated levels of IL-6 have been found in culture supernatant of cancer cells, especially, multidrug resistant cell lines and CSCs enriched culture [103–106]. Accordingly, IL-6 receptors are expressed by large range of cancer cells [107, 108], while in the physiological conditions they are only expressed on activated B cells, resting T cells and hepatocytes [109].

IL-6 is involved in the proliferation, differentiation and apoptosis resistance of various malignant tumor cells, such as multiple myeloma [110, 111], lung cancer [112], renal cell carcinoma [113, 114], breast cancer [115, 116], colorectal cancer [99, 117], endometrial cancer [118], cervical cancer [119], and ovarian carcinoma [120, 121]. Overexpression of both IL-6 and its receptors (IL-6R and sIL-6R) is common in breast carcinoma [115], oral squamous cell carcinoma [122] and prostate cancer [123]. In addition, tumor clinical samples containing significantly higher levels of IL-6 protein compared to those of control tissues and a higher IL-6 mRNA, correlate with poor patient survival.

IL-6 has been also found to play a central role in cancer dissemination and invasion through VEGF synthesis stimulation and this, consequently, enhances tumor vascularization and angiogenesis. Previous studies demonstrated a poorer overall survival (OS) and progression-free survival (PFS) of colorectal cancer patients with high IL-6 serum levels compared to patients with low IL-6 serum levels. Results of patients with colon cancer demonstrated that the serum IL-6 level but not the plasma VEGF level affected the response to the anti-angiogenic antibody bevacizumab. However, tocilizumab, an anti-IL-6 receptor antibody, suppressed tumor progression and angiogenesis. Two hypotheses were suggested, IL-6 can either promote the secretion of angiogenic factors that might affect bevacizumab efficacy, or mediate VEGF secretion by cancer-associated fibroblasts (CAF) [124].

Finally, IL-6 plays a pivotal role in cancer cachexia and the hypercatabolic state produced by this cytokine causes several symptoms such as anorexia, lowered serum albumin, a reduction in hemoglobin and a decrease of body mass index [125].

## IL-6 and cancer cell stemness properties

In cancer stem cells studies, IL-6 has been considered as a key regulator on CSCs self-renew. Wan et al. revealed that IL-6 promotes the expansion of human hepatocellular carcinoma (HCC) stem cells. IL-6 mRNA was detected in 73% of HCC patients and significantly correlated with the expression of CD44 (an important CSCs marker), POU5F1, HIF-1α, β-catenin, Snail, and HEY1 [126]. The correlation between IL-6 expression and HCC stemness was also supported by the analysis of a publicly available HCC patients gene expression data set [127]. The study also suggested that IL-6 was the most significantly increased cytokine in the supernatants of HepG2/TAM co-cultures by examination of an array for 82 cytokines/chemokines. ELISA results confirmed that HepG2 cells alone did not produce IL-6 and TAMs alone showed low IL-6 levels, whereas HepG2/TAM co-cultures had a more than 10-fold increase in the expression level of IL-6, and a qPCR analysis indicated that this IL-6 was mainly produced by TAMs. In the same experiment, the addition of IL-6 to HepG2 or Hep3B cells increased CD44-expressing cells population and sphere formation capacity [128].

Furthermore, Wang et al. using immunofluorescent staining of frozen sections of human glioma surgical biopsies found that IL-6 receptors and CD133 were coexpressed in freshly isolated glioma simples. Targeting IL-6R or IL-6 ligand gp130 expression in glioma stem cells with a shRNA significantly reduced growth and neurosphere formation capacity, at the same time increased the apoptosis in GSCs culture and survival of mice bearing intracranial human glioma xenografts. A decreased growth of subcutaneous human GSC-derived xenografts treated with an IL-6 antibody was also observed in mice. The study suggested a paracrine origin of IL-6 from the surrounding stroma cells, in addition to the autocrine production from the cancer cells themselves [129]. IL-6 downstream pathway Jak/Stat3 was found to be involved in GSCs self-renewal. Permanent loss of stem cell characteristics was observed when Stat3 is blocked and treatment with Stat3 inhibitor impressively inhibits tumor formation.

IL-6 signaling has also been found to play an important role in breast cancer stem cells -or so-called mamospheres-progression and survival. High levels of IL-6 mRNA were observed in MCF-7 mamosphere-enriched culture and in mamosphere isolated from node invasive breast carcinoma tissues. It has been observed that the administration of 10 ng/ml of IL-6 increased the secondary mamospheres formation in normal and tumor mamospheres derived from the same patient (self-renewal capability), while the treatment of mamospheres with an anti-IL-6 antibody abolished the self-renewal capability [130]. Sansone et al.showed that IL-6 acted on stem cells by targeting *Jagged-1* gene, which belongs to a family of Notch ligands, and *CA-IX*, a hypoxia survival gene leading to a Notch-3–dependent ERK activation. These suggested that IL-6 might trigger a potential autocrine/paracrine Notch-3/Jagged-1 loop to boost stem/progenitor self-renewal in the mammary gland. Together, these data indicate that IL-6 signaling contributes to cancer malignancy through the promotion of CSCs growth and survival, and that targeting IL-6 may offer benefits for hepatoma, glioma and breast cancer patients.

## IL-6 targeted therapy

Based on the rich body of studies on the biological activities of IL-6 and its pathological roles, therapeutic strategies targeting IL-6 signaling pathway are in large development for cancer, inflammation and autoimmune diseases.

## Monoclonal antibodies

Tocilizumab (TCZ, Actemra®), an anti-IL-6 receptor antibody, is effective in the treatment of various autoimmune and inflammatory conditions notably rheumatoid arthritis, and it is the only anti-IL-6 mAb that has been approved in clinic. It has been used in Japan since 2005 to treat patients with Castelman’s disease and arthritis. Tocilizumab showed the capacity to inhibit the growth of U87MG glioma cell [131], and suppressed the in vivo growth of human oral squamous cell carcinoma (OSCC) by inhibiting IL-6/STAT3 [132] and angiogenesis [133] signaling pathways. Tocilizumab inhibited lymph node metastasis, which was associated with resistance to conventional therapy and poor survival of patients [134]. This antibody is now being evaluated in open-label phase I (USA) and phase II (France) clinical trials as a monotherapy for melanoma patient’s treatment. Tocilizumab showed also an ameliorative effect on cachexia induced by metastatic lung cancer, where the survival rate was prolonged for 9 months without any chemotherapy in a reported case [125].

Siltuximab (CNTO 328), an anti-IL-6 antagonist antibody developed by Janssen Biotech possesses potential benefits in treating various human cancers either as a single agent or in combination with other chemotherapy drugs. In a clinical study, prolonged periods of disease stabilization in ovarian cancer patients with recurrent and drug-resistant diseases were observed after treatment with siluximab. In phase I/II trials in metastatic renal cancer, it stabilized the disease in more than 50% of patients [135]. No adverse effects were observed in a phase I study of prostate cancer [136] and its use in combination with docetaxel appears to be safe and shows an efficacy in patients with castration-resistant prostate cancer. Siltuximab has been reported to reduce cancer-related anorexia and cachexia and neutralize the effect of IL-6 in different types of human malignancies.

Other anti-IL-6 mAbs are in development and have shown promising effect in the treatment of several cancer type (Table 2). mAb 1339 (Azintrel OP-R003), a fully human version of the murine mAb, could inhibits the growth of melanoma cells in the presence of bone marrow stromal cells in vitro. In a SCID-hu mouse model of melanoma, mAb 1339 significantly enhanced the function of dexamethasone in vivo [137]. Elsilimomab (BE-8) [138, 139], murine monoclonal antibody has been also developed and examined in preclinical studiesAnother anti-IL-6 antibody, clazakizumab (BMS945429, ALD518), originally used in inflammatory diseases treatment, has been tested for NSCLC-related fatigue and cachexia treatment. [140]. Completed Phase IIa clinical trial showed that it improved anemia and lung symptom score, reversed fatigue and loss of body mass. Another phase II randomized clinical trial is ongoing to evaluate the safety and efficacy of clazakizumab for reducing oral mucositis in head and neck cancer patients. Finally, the mAb sarilumab showed a good effect both as a single agent or in combination with aflibercept, an inhibitor of VEGF [141]. However a case report showed a complete remission in a patient with relapsed refractory multiple myeloma after single agent therapy with siltuximab [142]. Clinical trials for the treatment of advanced multiple myeloma, hormone-refractory prostate cancer, ovarian cancer, non-Hodgkin’s lymphoma, and renal cancer have been discontinued due to lack of efficacy [143].

**Table 2:** Summary of clinical approaches for inhibiting IL-6 activity in cancer

Class	Drug	Type	Clinical trials	Ref.
Monoclonal antibodies	Tocilizumab	Humanized anti-IL-6R mAb	Cancer cachexia in lung cancer and phase I + II for multiple Myeloma	[125]
	Siltuximab	Chimeric anti-IL-6 mAb	Phase II multiple myeloma, phase I completed for B-cell non-Hodgkin’s lymphoma, multiple myeloma, phase II prostate cancer, phase II renal cell carcinoma, phase I oral mucositis in head and neck cancer and phase II non-small cell lung cancer-related cachexia	[135]
	Clazakizumab	Humanized, aglycosylated anti-IL-6 mAb	Multiple myeloma and phase II renal cell carcinoma	[140]
	Elsilimomab (BE-8) Siltuximab	Murine anti-IL-6 mAb Chimeric anti–IL-6 mAb	Multiple Myeloma Clinical trial for multiple myeloma, hormone-refractory prostate cancer, ovarian cancer, non-Hodgkin’s lymphoma, and renal cancer has been discontinued due to lack of efficacy	[138, 139] [141]
Non-mAbs agents	sgp130Fc	Fc fusion gp130 IL-6 signaling blocker	Preclinical studies	[194]
	SANT-7	IL-6R antagonist	Combination therapy in multiple myeloma	[195]
	ERBA	small molecule IL-6R antagonist	Cancer-related cachexia	[196]
	Atiprimod	Small molecule JAK2 and JAK3 inhibitor	Clinical trial in development for multiple myeloma	[195]
	Ruxolitinib	Small molecule JAK1 and JAK2 inhibitor	Phase II clinical trial for myeloproliferative neoplasms (MPN) and AML	[146]

## Non-mAbs agents

Several anti-IL-6-based therapies are also under clinical development, sgp130Fc, a novel blocker of IL-6 signaling, consists of gp130 fraction linked to IgG-Fc [144]. SANT-7 is an IL-6Ra and ERBA is a non-peptide IL-6Ra. Some small molecule compounds inhibiting IL-6 and its downstream targets have also been developed and evaluated in preclinical and clinical studies of various human cancers [145]. These small molecules include atiprimod [145]; ruxolitinib [146]; and the JAK2 inhibitor CEP-33779 [146]. New strategies, such as combination of IL-6 blocking and inhibition of other signaling pathways, might further improve IL-6-targeted immunotherapy.

## Il-4

IL-4, the most important Th2 cytokine, is mainly produced by activated T cells, mast cells, basophils, and eosinophils to regulate lymphocytes proliferation and survival. Besides mediating its biological functions, IL-4 could also promote the proliferation and survival of several cancer cells. It was found to be over expressed by many human tumor types including malignant glioma, ovarian, lung, breast, pancreatic, colon, and bladder carcinomas, which also overexpress its receptors (IL-4R) [147–149]. IL-4R is composed of several subunits. Depending on the cell type, diverse binding configurations generate different types of IL-4/IL-4R complexes [150, 151]. The type II IL-4R, composed of the two subunits IL-4Rα and IL-13Rα1, is the predominant complex in cancer cells [152]. The receptor complex initiates signal transduction through JAK/STAT6 pathways [153]and could also signal through IRS/PI3K/AKT pathway [154, 155]. IL-4 and IL-13 stimulation activates STAT6 that binds to promoters of various genes and plays an important role through the modification of cell differentiation and growth, colonization induction and apoptosis resistance. It is very interesting that mice lacking STAT6 manifest enhanced tumor immunity to both primary and metastatic mammary carcinomas [156, 157], and induce spontaneous rejection of implanted tumors. Furthermore, cells defective in this pathway exhibit increased spontaneous apoptosis in vitro [158]. This strongly supports the hypothesis that IL-4/STAT6 signaling may be beneficial to tumor growth possibly by several mechanisms, including gaining resistance to apoptosis and escaping the immune surveillance [159].

Acute tumor-directed immune responses involving the Th1 CD4^+^T-helper cells enhance antitumor immune responses by INFγ secretion, which in turn induces activation of macrophage cytotoxic activity and, consequently, inhibits tumor development [160]. In contrast, immune responses involving chronic activation of humoral immunity and Th2-polarized CD4^+^ T-helper cells that express IL-4. The expressed IL-4 induces T-cell anergy and loss of T-cell-mediated cytotoxicity leading to the promotion of tumor development and disease progression [161].

## IL-4 and cancer

**Fig. 5:**
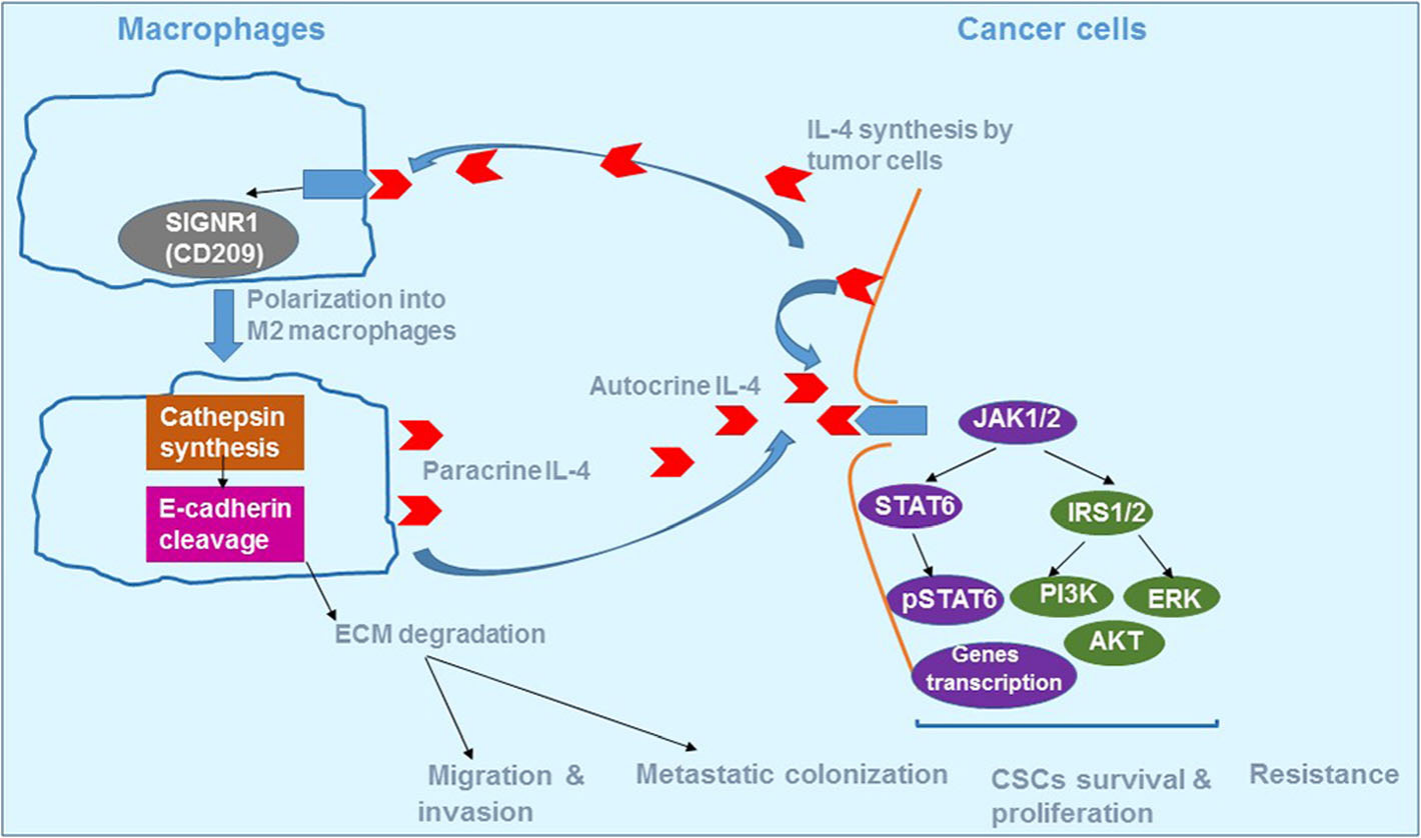
IL-4 induces the polarization of macrophages recruited in the tumor microenvironment through the SIGNR1 activity. The polarized macrophages are characterized by high levels of cathepsin protease activity, resulting in promoting tumor growth, angiogenesis, and invasion. Both paracrine and autocrine IL-4 activates the identical intracellular pathways generating pSTAT6, ERK, and AKT leading to tumor invasion, metastasis, resistance and CSCs survival

The autocrine origin of IL-4 is now believed to be an indicator of tumor aggressiveness. The IL-4 secreted by cancer cells is responsible for tumor-associated macrophages (TAM) polarization toward M2 macrophages inducing cathepsin activity (Fig. 5). High cathepsin proteases activity was observed in the majority of macrophages in the microenvironment of pancreatic islet cancers, mammary tumors, and lung metastases during malignant progression; and it is critical for promoting tumor growth, angiogenesis, invasion and, likely, tumor metastasis. The proposed mechanism was that E-cadherin is cleaved by cathepsins B, S, and L to initiate loosening of cell–cell contacts, degradation of ECM substrates, and consequently generating pores for cancer dissemination [162, 163].On the other hand, using a Lewis lung cancer model, Yan et al. claimed that IL-4-induced macrophages polarization is probably mediated by SIGN (CD209) expression [164].

The paracrine origin of IL-4 from the cells resident in tumor microenvironment have also been reported. Different Th2 cytokines level assessment in the serum of patients with breast cancer, revealed a remarkably increased IL-4 in hormone-receptor negative tumors, and was related with patients’ death from cancer [165]. Further research supported the important role of IL-4 in the expression of breast cancer related hormones, specifically estrogen [166]. Other work reported a significantly higher IL-4 level in node-positive breast cancer patients [167–169]. Razmkhah et al. suggested that resident adipose derived stem cells (ASCs) in breast cancer tissue could express IL-4 and the mRNA expression of IL-4, which contributes with IL-10, TGF-b1, CD25 and CCR4, in increasing the metastasis of breast cancer cells and the recruitment of Tregs to protect cancer cells from the host immune response [170]. Moreover, Il4/IL4R signaling trough Stat6 has been shown to promote the survival and proliferation of breast and colon cancer in a nude mice model, mainly via the upregulation of anti-apoptotic proteins PED, cFLIP, Bcl^−^xL and Bcl^−^2. A study on prostate cancer showed elevated IL-4 level in patients with hormone-refractory cancer. Moreover, recombinant IL-4 application also upregulated the expression of the two proteins - annexin A5 and syncytin that play important roles in several cell-cell fusion processes. IL-4 inhibition, indeed, lowers their expression and suppresses cell proliferation and fusion [171, 172]. In pancreatic tumor, IL-4 autocrine origin is essential in the control of normal macrophages transition into tumor-promoting macrophages. IL-4 expression was low in normal islets and increased by 4.5-fold at the hyperplastic stage. However, upon induction of angiogenic switching, IL-4 mRNA levels were increased by 7.7-fold in angiogenic islets and 7.9-fold in end-stage tumors. In addition, the evaluation of the cytokines present at primary renal cell cancer site by PCR for freshly isolated RCC TIL, as well as established RCC lines, revealed an increased IL-4 mRNA level in freshly collected RCC TIL and short-term-cultured RCC tumor lines [172].

In addition to cell proliferation and survival, IL-4/IL-4R signal plays a key role in tumor metastasis. Using a shRNA-mediated knockdown (K_D_) of IL4Rα mice model, Venmar et al. proved that IL-4 can enhance cancer metastatic and outgrowth in the lung and liver [173]. In support of this, systemic neutralization of IL-4 using a monoclonal antibody in a murine spontaneous mammary cancer model was shown to reduce tumor lung metastasis. This study suggested that the IL-4 produced by CD4^+^ cells upon a chronic activation, promotes the polarization and pro-tumor bioactivity in macrophages, that in turn enhance tumor cells malignancy and metastasis [174].

Furthermore, IL-4 contributes in MUC2 regulation in colon cancer and its addition to cancer cells increases MUC2 secretion through a MAP kinase pathway, leading to the so-called mucinous carcinomas [175].

## IL-4 and cancer cell stemness properties

Several studies have revealed a close relationship between IL-4 and cancer cell stemness properties. In breast cancer, both autocrine and paracrine IL-4 production regulates BCSCs (breast cancer stem cells) features, including cell proliferation, motility and cytoskeletal organization via the RAS/MAPK pathway. Interestingly, blocking IL-4 suppressed BCSCs proliferation, colony forming efficiency and in vivo tumor formation, while it supported the expression of the dual specificity phosphatase-4 (DUSP4) in triple-negative basal-like BCSCs, leading to aggressiveness reduction [172]. Moreover, treating prostate cancer cells with recombinant IL-4 resulted in a robust fusion between cells and a significant increase in the percentage of cells expressing stem cell marker CD133, drug resistance properties, mesenchymal-characteristic markers pAKT, MMP-9, desmin expression, and loss of E-cadherin [171].

IL-4 signaling may offer a new therapeutic tool in colon carcinoma. IL-4 dependent resistance of colon cancer cells is likely a tumor autocrine process that establishes an anti-apoptotic program in cancer stem-like cells, and protects them from chemotherapy [176].

## IL-4-targeted therapy

Several therapeutics have been developed for asthma and inflammatory diseases treatment, but the non-efficacy has stopped their development. However, most of the therapeutics showed a good tolerance and a high safety in humans, making the development of clinical trials for cancer therapy a possibility (Table 3).

**Table 3:** clinical trials for IL-4 inhibition in cancer

Class	Drug	Effects	Clinical trials	Ref.
Targeting signaling	Ruxolitinib leflunomide	Inhibition of JAK1/2 resulting in STAT6 phosphorylation inhibition Inhibition of STAT6 Phosphorylation	FDA approved for myelofibrosis. Phase I/II trials for CML, AML, colorectal cancer, NSCLC, lung adenocarcinoma, and breast cancer in preparation. Approved for RA treatment, immunosuppressive therapy after organ transplantation	[178, 197] [144, 198]
Blocking IL-4/IL-4R interaction	Dupilumab AMG317 Pitrakinra Altrakincept Pascolizumab	Humanized anti–IL-4Rα mAb Humanized anti–IL-4Rα mAb modified form of IL4 Recombinant human IL4R Humanized anti–IL4 (IgG1) Antibody	Phase III trials for asthma and atopic dermatitis are ongoing Good results of Phase I/II trials in asthma then abandoned due to lack of efficacy Completed phase I/II/III trials in asthma. Abandoned after Phase I/II trials completion for asthma due to lack of efficacy Abandoned after Phase I/II trials completion for asthma due to lack of efficacy	[182, 199] [185] [185, 200] [184, 201] [202]

## Targeting signaling pathways

Targeting JAK1/JAK2 leads to the inhibition and suppression of the first step of IL-4 signaling pathway. Taking in consideration that JAKs signaling is not specific to IL-4, and mediates signaling response for a variety of cytokines, this strategy would not be the most suitable signaling blocking mechanism. However, several JAK inhibitors exist for the treatment of asthma and other inflammatory diseases, and some are in clinical trials for cancer treatment [177]. Ruxolitinib, approved by the FDA for myelofibrosis, is the most known JAKs inhibitor; several trails on hematopoietic and solid tumors are in development to test its action in blocking the IL-4/IL-4R interaction [178].

STAT6 may be another nonspecific target for IL-4 pathway and its blockade would result in the attenuation of both IL-4 and IL-13 signaling in immune and non-immune cells, with non-well-known implications. There are two FDA approved therapies known to inhibit STAT6 phosphorylation, leflunomide and vorinostat. Their applications in cancer therapy remains very limited because of their multiple modes of action (de novo pyrimidine synthesis and tyrosine kinases inhibition [179], and histone deacetylases inhibition [180], respectively). Another signaling pathway targeting possibility is AKT and ERK inhibition [181], but the diversity of their role hinders its development.

## Blocking IL-4/IL-4R interaction

In this strategy, the blockade could be achieved either by inhibiting IL-4R or by neutralizing the IL-4 present in the tumor environment. Dupilumab, a monoclonal antibody against IL-4Rα developed and tested in clinical trials, has shown efficacy in atopic dermatitis [182], and asthma treatment [183]. AMG317, a humanized IL4Rα antibody was abandoned after phase II clinical trial because of the non-efficacy in asthma treatment [184]. Pitrakinra, or IL-4 double mutein developed for asthma treatment [185]. Altrakincept and pascolizumab are two mAbs neutralizing IL-4. They were initially developed and tested for asthma treatment but their development was halted after phase II clinical trial because of the non-efficacy [186].

## Conclusions

Interleukins constitute a substantial proportion of the cytokines within the tumor microenvironment. They are classified among the best-validated therapeutic targets in anti-cancer immunotherapy. Data provided in this review demonstrate that most proinflammatory cytokines (IL-1, IL-4, IL-6) produced by either host immune cells or tumor cells themselves are associated with tumor malignancy in patients and animal cancer models. In contrast, anti-inflammatory cytokines (IL-2, IL-15, and IL-21) usually activate the antitumor immunity and interfere with tumor development. These evidences provide a therapeutic strategy based on selective interference with the proinflammatory and tumor-promoting cytokines action to prevent the pro-survival and growth-promoting effects, while enhancing the activity of anti-inflammatory interleukins renders the cancer cells more susceptible to the elimination by host immune cells.

## Abbreviations

CAF: Cancer-associated fibroblasts
CAPS: Cryopyrin-associated periodic syndromes
CSCs: Cancer stem cells
CTLA-4: Cytotoxic T-lymphocyte associated antigen 4
CTLs: Cytotoxic T lymphocytes
DCs: Dendritic cells
ECM: Extracellular matrix
HCC: Human hepatocellular carcinoma
HIF-1α: Hypoxia inducible factor
IL: Interleukin
JAK: Janus family tyrosine kinases
LAK: Lymphokine-activated killer
mAbs: Monoclonal antibodies
MAPK: Mitogen-activated protein kinase
MEK: Mitogen-activated protein kinase kinase
MMP: Matrix metalloproteinase
MPE2: Mammaloglobin promoter/enhancer
NK: Natural killer
NSCLC: Nonsmall-cell lung cancer
OPG: Osteoprotegerin
OS: Overall survival
PD-1: Programmed death 1
PFS: Progression-free survival
PI3K: Phosphoinositide 3- kinase
RCC: Renal cell cancer renal cell cancer
SHP2: Src homology 2 domain containing tyrosine phosphatase 2
STAT: Signal transducer and activator of transcription
TAM: Tumor-associated macrophages
TCR: T-cell receptor complex
TILs: tumor infiltrating lymphocytes
TNBC: Triple-negative breast cancer cell lines.
